# Knowledge, perception and practice of injection safety and healthcare waste management among teaching hospital staff in south east Nigeria: an intervention study

**DOI:** 10.11604/pamj.2014.17.218.3084

**Published:** 2014-03-19

**Authors:** Oguamanam Okezie Enwere, Kevin Chiekulie Diwe

**Affiliations:** 1 Department of Internal Medicine, Faculty of Medicine, Imo State University, Owerri, Nigeria; 2 Department of Community Medicine, Faculty of Medicine, Imo State University, Owerri, Nigeria

**Keywords:** Needle prick, post-exposure prophylaxis, colour coding, health workers, teaching hospital, South Eastern Nigeria

## Abstract

**Introduction:**

Health care workers are exposed to the risk of blood-borne diseases such as HIV, Hepatitis B and C in their daily encounter with infected patients and materials through unsafe injections. This study determined the baseline and post-intervention knowledge and practice of modern injection safety standards among health care workers.

**Methods:**

The study population was the healthcare workers in a teaching hospital in southeastern Nigeria. Data was collected using a self-administered 37-item structured questionnaire assessing their knowledge and practice on injection safety. Collected data was analyzed using SPSS.

**Results:**

Nurses comprised 62.8% (98/156) of the population. While most had heard of injection safety only 67.2% (84/125) had previously had any form of training on it. Only 54% (81/150) had heard or seen color coded bins. The standard needle and syringe is still widely used and 45% (65/145) still recap needles on syringes after use irrespective of type of personnel. Half (50.6% =78/154) of our respondents had had a previous needle prick injury. Only 25.6% (20/78) with previous needle prick injury had post-exposure prophylaxis. All doctors and laboratory scientists always use gloves compared to 94.8% (91/96) nurses while handling patients or materials. Following the intervention, a significantly high number became aware of post-exposure prophylaxis and color coded bins and liners.

**Conclusion:**

There is a need for healthcare workers to be regularly updated on changing safety standards in their practice. Also hospitals must be encouraged to acquire and use internationally accepted standard materials in collection and disposal patient's samples.

## Introduction

Work environments have peculiar health-related risks. Doctors and other healthcare workers (HCWs) encounter work-related hazards associated with their clinical and laboratory activities in the hospital [1, 2]. Blood and other effluents from infected patients may bear such pathogens as HIV, hepatitis B and hepatitis C virus, increasing the risk of transmission from such accidents as needle pricks and contacts with deep body fluids [3, 4]. A safe injection is one that is given using appropriate equipment; does not harm the recipient, does not expose the provider to any avoidable risk and does not result in any waste that is dangerous to other people (the community) [3, 4].

Injection safety is an integral component of infection prevention and control; an element of standard precautions; key element of patient and healthcare worker safety; supported by infection prevention and control policies and procedures such as hand hygiene, housekeeping and waste management; a critical item of the continuous quality improvement (CQI) programme, managed by the healthcare team and specifically the infection prevention and control team in the health facilities.

Unsafe injection practices are associated with risks such as: (a) Transmission of infections e.g. HBV, HCV and HIV; (b) Paralysis resulting from injection of a drug into a nerve resulting in weakness of the limb supplied by the nerve; (c) Adverse events following injections of which the most threatening is anaphylaxis (Sudden collapse of the circulatory system due to immunological response to the injected drug). The groups at risk of these unsafe injection practices include: (a) Patients / clients especially immune-compromised individuals; (b) Healthcare workers especially doctors, nurses, laboratory scientist, laundry worker etc.; (c) Healthcare waste management personnel; (d) Communities through indiscriminate healthcare waste disposal; (e) Drug users.

The World Health Organization (WHO) estimates that 16 billion injections are administered annually in developing countries of which 90-95% is for therapeutic purpose. Approximately 5% of HIV, 40% of Hepatitis C and 32% of Hepatitis B virus infections are caused by unsafe and unnecessary injections worldwide [3, 4]. According to a national cross-section survey conducted in August, 2004, disposable syringes and needles have been used almost exclusively since the 1980s in Nigeria. However a high percentage of the facilities were discovered not to always adhere to injection safety practices. The WHO defines healthcare waste as the total waste stream from a healthcare or research facility that includes both potential risk waste and non-risk waste materials.

Risks and hazards associated with healthcare waste include: needle stick injuries; transmission of infections or diseases; re-use of some types of waste (accidental or intentional); environmental pollution or degradation; exposure to radiation; fires and public nuisance (Offensive smells, unsightly debris). Eighty percent (80%) of healthcare waste is general waste or low risk waste, 20% can be dangerous and referred to as risk waste while 1% of risk waste is sharps waste [3, 4].

Waste bins are containers that are made of non-corrosive materials, leak proof with well fitting lids, washable and portable. These waste bins as well as their bin liners are colored black, red, yellow, brown, yellow with biohazard sign for disposal of the various categories of healthcare wastes. Waste minimization, segregation, handling and storage, transportation, treatment and destruction before final disposal are the steps involved in healthcare waste management (HCWM). Proper healthcare waste management will minimize/eliminate the hazards associated with healthcare wastes.

This study aimed to assess the knowledge, perception and practice of injection safety and HCWM is important as increasing the knowledge and positively changing the attitude and practice of injection safety and HCWM through training of healthcare workers will go a long way in minimizing/eliminating the above mentioned risks associated with unsafe injection practices and improper HCWM.

It has been observed that the highest prevalence of HIV-infected persons is found in developing countries and the highest needle prick injuries are also recorded in this region of the world [5]. Injuries from needle pricks are thought to be the commonest work-related hazard reported from a Nigerian teaching hospital [6]. The risk of acute hepatitis C infection in a health worker following a needle prick injury has been estimated to be from 1% to 5% [7]. The available estimates suggest that contracting hepatitis B infection due to a needle prick injury has a risk close to 100 times that of contracting HIV [8]. Needle prick injuries are a common occurrence among health workers in Nigeria. It is estimated that more than 80% of workers in the health care sector will experience a needle prick injury at some point in their career [9], and this is commoner amongst the surgeons, house officers and younger nurses [9, 10]. Recently, the standard needle and syringe (disposable) is being replaced with safer alternatives (auto-disable syringes, vanish-point needles while improvements are being made for safer disposal of wastes using color coded bins and bin liners.

Imo State University Teaching Hospital (IMSUTH) is an Institute of Human Virology of Nigeria (IHVN) accredited site for management of People Living With HIV/AIDS (PLWHA). The Institute organized a Training of Trainers (TOT) workshop on injection safety in which the second author with another staff of this hospital participated.

In collaboration with Institute of Human Virology of Nigeria (IHVN), this hospital organized a three (3) day training workshop on injection safety and HCWM for a cross-section of the hospital staff aimed at improving injection safety practices and HCWM and embracing internationally accepted safety practices in the institution. The study assessed the pre-training knowledge and practice of injection safety among the participants and also assessed the impact of the post-training on them.

## Methods

The study was part of a three (3) day training workshop designed at educating the health workers in various departments of the hospital on the modern universal safety methods as well as the materials available to achieve optimum safety of health care workers when handling patients and their products like blood, body fluids and feces.

The study location was the Imo state University Teaching Hospital, Orlu, Imo State. Orlu is a semi-urban town with an estimated population of two hundred and twenty thousand (220,000) [11]. The teaching hospital is a 200-bed facility that serves as a training institution for medical students, nurses and laboratory scientists; as well as serving as a referral center for tertiary level health care to the people of Orlu and its environs.

The study population comprised doctors, laboratory staff, nurses, orderlies & waste handlers representing the various wards and theatre nurses. These were to serve as the initial trainees who will eventually step down the gained knowledge to their colleagues. Also some resident doctors representing the various departments and laboratory staff participated in the program.

The study instrument was a 37-item structured questionnaire that assessed the health workers’ baseline knowledge of injection safety; their practice in relation to injection safety and their perception of its practice. The questionnaire was adapted from the one used during the Training of Trainers workshop done in 2009 on injection safety and HCWM. And pre-tested at another tertiary heath institution before use in this study. The questionnaire was self-administered to the literate staff and interviewer-administered to the semi-literate ones like waste handlers and nurse assistants after translation to the local language (Igbo).

The instrument was initially administered before the training on injection safety commenced on day 1 at IMSUTH. The same instrument was again administered after the training program and a comparison with the initial assessment done to identify the impact of the training. The self-administered questionnaires were immediately collected on completion. It took approximately 15 minutes to complete. No names were required from participants to encourage anonymity, and participation was voluntary.

Data collected was entered into a password protected computer and analyzed using SPSS 15.0. Data is presented in tables, figures and proportions. Ethical approval was obtained from the teaching hospital Ethics committee. The limitations of the study were as follows: the recall bias could have occurred due to the reliance on self-report by the respondents, and the sample was from the staff that are at greater risk of exposure.

## Results

One hundred and fifty six (156) people participated in the pre-training test while 139 participated in the post-training test (attrition rate of 10.9%).

**Pre-training:** The demographic distribution of the participants is shown in Table 1. Median age of participants was 35 years (range 20-54 years). Mean age for the females was 36.3 years (±7.2) compared to the males 35.8 years (±6.9) (P= 0.75). The average time for which the participants had been working was 9.6 years (±8.2), median 5 years (range 6months -34 years).

**Table 1:** Demographic distribution of respondents

Demography						Total
Sex	Female =125 (80.1%)	Males =31 (19.9%)				156
Status	Nurses =98 (62.8%)	Ward assistant =19 (12.2%)	Waste handlers =18 11.5%)	Doctors =13 8.3%)	Lab scientists =8 (5.1%)	156

**Injection safety knowledge:** As many as 125 (81.7%) had heard of injection safety; of this number 67.2% (84/125) had had some training on it but only 72 (46.2%) had formal training on injection safety. About 88.7% (134/151) thought injection safety was of any benefit to the health worker; 86.6% (123/142) thought a safe injection does not expose a health worker to avoidable risks. Only 68% (104/153) knew of post-exposure prophylaxis while 54% (81/150) knew of or had seen color coded bins; a larger proportion 81.2% (121/149) knew of safety boxes. Only 43.9% (54/123) think the standard syringe and needle should be abandoned.

**Injection safety practices:** Table 2 shows some of the practices observed. Less than half of the respondents do recap needles on syringes after use with no difference in the status of the respondent (χ^2^ =1.82, p= 0.77). Most commonly used syringe in the hospital was the standard disposable syringe & needle (82.9%); the manually retractable and automatically retractable ones were used sometimes (7.2% and 6.6% of the time respectively). A third, 66.7% (92/138) believe wastes should be disposed in a special way. The largest proportion of those with previous NPI were doctors (66.7%) compared to nurses (52.6%), waste handlers (50%), orderlies (42.1%) and lab scientists (25%) (χ^2^ =4, p= 0.4). Also the largest proportion of healthcare providers who had a needle stick injury in the last one year were doctors (50% of them). Only 30.7% (42/137) knew there was a hospital needle-prick injury accidents management (NPIAM) protocol and register for post-exposure prophylaxis.

**Table 2:** Injection safety practice among respondents

Some observed practices	Yes	Number
Recap of needles	44.8%	65/145
Separation of hospital wastes	50%	74/148
Previous needle prick injury	50%	74/148
Obtaining NPIAM or PEP following a NPI	26.7%	20/75
Routine use of gloves	92.2%	141/153

All doctors and laboratory scientists always used gloves compared to 94.8% (91/96) nurses, 88.9% (16/18) waste handlers and 73.7% (14/19) orderlies. While all waste handlers do not use industrial gloves when handling wastes, only 16.7% (3/18) use boots while working. Over half (58.2% = 89/153) of the respondents routinely use an apron/overall while working with patients requiring a procedure. This was significantly higher among the doctors, nurses and laboratory scientists compared to the orderlies and waste handlers (χ^2^ =14.7, p= 0.005). 56.2% (86/153) of the respondents routinely use face masks while handling wastes. While 66.7% and 64.6% of the doctors and nurses use face masks, 50%, 36.8% and none of the waste handlers, orderlies and laboratory scientists respectively use face masks.

### Perception (Table 3)

**Post-training:** Seventeen respondents comprising 16 nurses and one ward assistant did not participate in the post-training assessment due to their work schedule. In the post test, though a larger proportion of respondents believe that injections safety is beneficial to health workers it was not significant (94.1% to 88.7%, OR 0.5, CI 0.2-1.2). This is also similar with the perception of benefit to the patient and community (92% to 88.6%, OR 0.67, CI 0.3-1.6) and (91.3% to 88.6%, OR 0.74, CI 0.3-1.7) respectively. No difference was observed on whether respondents thought a safe injection does no harm to the recipient, was of any danger to the community or whether it was environmentally friendly (χ^2^ =2.2, p= 0.33; χ^2^ =1.6, p= 0.44; χ^2^ =0.9, p= 0.64 respectively). A significantly higher number were aware of post-exposure prophylaxis (82.4% to 68%, OR 0.5 CI 0.3-0.8) and similarly larger proportions were now aware of color-coded bins and liners (80.1% to 54%, OR 0.29, CI 0.2-0.5) and safety boxes (93.4% to 81.2%, OR 0.3, CI 0.1-0.7). Also a larger proportion believe the standard syringe should be stopped (59.7% to 43.9%, OR 0.5, CI 0.3-0.9).

**Table 3:** Injection safety practice among respondents

Perception	Yes	Number
Awareness of hosp waste management team	6.1%	9/147
Hospital waste should be disposed specially	95.4%	144/151
Preference of oral to parenteral medications	94.5%	138/146
Appreciation of training on injection safety	96.6%	144/149
Belief that injection safety is protective	95.2%	138/145

## Discussion

Injection safety practices are becoming very necessary in view of the rising awareness in the associated risks involved such as transmission of HIV and hepatotropic viruses. Such efforts as the use of retractable needles and safe disposal of hospital wastes as well as use of personal protective measures cannot be over emphasized. With about half of the study population having had any formal training on injection safety and also lacking awareness of post-exposure prophylaxis reflects the poor knowledge base of these workers.

Our study suggests that personal safety measures were poorly adhered to when handling patients generally, with over half of the respondents having had needle prick injuries in the past. In Nigeria poor adherence to safety practices and considerably little experience among medical personnel contribute to the risk of NPIs [10–12]. Also noted in our study was the higher proportion of NPIs among doctors compared to other HCWs, a finding similar to the observation made by Sadoh et al [13]. Doctors, who by virtue of their long and higher level of educational training are supposed to know and practice better, tend to exhibit less caution in potentially dangerous situations.

It is alarming to note that only a quarter of those who had had a NPI had NPIAM or PEP despite the fact that about two thirds of respondents knew of PEP and the hospital keeps a PEP register. It is obvious that the majority of workers are unaware of NPIAM and that PEP is available to them in the hospital. An awareness and practice of NPIAM and PEP reduces risk of transmission of HIV infection.

The impact of the three day program was significant in raising awareness on the following issues: PEP, the color coding of the various bins as well the importance of using the retractable needles compared to the standard needle and syringe. No change in perception was observed though this was generally good before the training. Although it is thought that education concerning occupational hazards as NPI is unlikely to sufficiently achieve and sustain a change in practice, educational programs may also be required to address attitudes and age-long beliefs [14, 15]. A three day impact on the practice of our respondents may likely also be influenced by challenges encountered in developing countries such as the lack of appropriate and adequate consumables and personal protective gears [16].

## Conclusion

The level of knowledge was high but poor in Post-Exposure Prophylaxis and Needle-prick injury accident management (NPIAM) protocol and register for post-exposure prophylaxis. Practice of injection safety standards was low except for routine use of hand gloves. Post-intervention assessment showed significant improvement in PEP/NPIAM and color coded bins awareness. The hospital management needs to enshrine the practice of injection safety standards by providing the necessary consumables e.g. color coded bins and liners, auto-retractable syringes and needles, safety boxes etc. There is also need for regular update training of staff on the changing safety standards in their practice.

**Limitations of the study :** 1) The attrition rate of 10.1% (17/156) was largely due to the nurses having to attend to their shift duties and their inability to conduct the post-training test on them; 2)Recall bias; since respondents were required to remember what had been imparted to them at the training workshop; 3)This study only assessed a section of the hospital and comprising about a third of the clinical staff.
